# Epidemiology of the Use of Hemostatic Agents in Children Supported by Extracorporeal Membrane Oxygenation: A Pediatric Health Information System Database Study

**DOI:** 10.3389/fped.2021.673613

**Published:** 2021-05-10

**Authors:** Marianne E. Nellis, Ljiljana V. Vasovic, Ruchika Goel, Oliver Karam

**Affiliations:** ^1^Department of Pediatrics, New York Presbyterian Hospital – Weill Cornell Medicine, New York, NY, United States; ^2^Department of Pathology and Laboratory Medicine, Weill Cornell Medicine, New York, NY, United States; ^3^Division of Hematology, Oncology, Department of Internal Medicine, Southern Illinois University School of Medicine, Springfield, IL, United States; ^4^Division of Transfusion Medicine, Department of Pathology, Johns Hopkins University, Baltimore, MD, United States; ^5^Division of Pediatric Critical Care Medicine, Children's Hospital of Richmond at VCU, Richmond, VA, United States

**Keywords:** extracorporeal membrane oxygenation, platelet transfusion, plasma, antifibrinolytic therapy, critical illness, children, hemostasis

## Abstract

**Objectives:** Children supported by extracorporeal membrane oxygenation (ECMO) are at high risk of bleeding. Though practitioners often prescribe blood components and/or medications to prevent or treat bleeding, the utilization of these hemostatic measures in children is not well-understood. We sought to evaluate the use of hemostatic blood products (platelet, plasma and cryoprecipitate transfusions) and medications [aminocaproic acid, tranexamic acid (TXA) and Factor VIIa] in children supported by ECMO.

**Design:** Retrospective observational study using the Pediatric Health Information System (PHIS) database from 2011-2017.

**Setting:** Fifty-one U.S. children's hospitals.

**Patients:** Children (aged 0–18 years) supported by ECMO.

**Interventions:** None.

**Measurements and Main Results:** ECMO was employed in the care of 7,910 children for a total of 56,079 ECMO days. Fifty-five percent of the patients were male with a median (IQR) age of 0 (0–2) years. The median (IQR) length of ECMO was 5 (2–9) days with a hospital mortality rate of 34%. Platelets were transfused on 49% of ECMO days, plasma on 33% of ECMO days and cryoprecipitate on 17% of ECMO days. Twenty-two percent of children received TXA with the majority receiving it on the first day of ECMO and the use of TXA increased during the 6-year period studied (*p* < 0.001). Seven percent of children received aminocaproic acid and 3% received Factor VIIa.

**Conclusions:** Children supported by ECMO are exposed to a significant number of hemostatic blood products. Antifibrinolytics, in particular TXA, are being used more frequently. Given the known morbidity and mortality associated with hemostatic blood products, studies are warranted to evaluate the effectiveness of hemostatic strategies.

## Introduction

Critically ill children have a significant risk of developing clinically significant bleeding due to underlying organ dysfunction and possible bone marrow suppression and/or consumptive coagulopathies (1). Children supported by extracorporeal membrane oxygenation (ECMO) are substantially more likely to bleed from a variety of factors, including initial hemodilution upon cannulation, required anticoagulation, circuit-induced consumptive coagulopathy and platelet dysfunction (2, 3). In a cohort of 514 children supported by ECMO at eight children's hospitals in the US, bleeding occurred in over 70% of patients and was independently associated with mortality (4, 5). In addition, bleeding in children supported by ECMO has been associated with fewer ventilator-free days and hospital-free days (6).

In order to prevent or treat such bleeding, pediatric intensivists often prescribe blood components, such as plasma, platelets and/or cryoprecipitate. In the same cohort described above examining the use of plasma and platelet transfusions, only one-quarter of the ECMO days were free from the transfusion of either blood product (7). Small epidemiologic reports have estimated that children supported by ECMO receive on average 75 mL/kg of plasma transfusions and 90 mL/kg of platelet transfusions during their hospital stay (8). No large-scale studies on blood product utilization in this vulnerable patient population have been reported.

In addition, in part due to the known mortality and morbidities associated with the transfusion of plasma and platelets in critically ill children, practitioners also use pharmacologic treatments such as anti-fibrinolytics [primarily tranexamic acid (TXA) or aminocaproic acid] or factor concentrates (such as Factor VIIa). The use of some of these agents have been described in small adult cohorts (9), but have not been reported in children supported by ECMO.

We sought to evaluate the utilization patterns of hemostatic blood products (platelet, plasma and cryoprecipitate transfusions) and medications (TXA, aminocaproic acid, and Factor VIIa) in children supported by ECMO using a large administrative database.

## Methods

We conducted a retrospective cohort study of children from the Pediatric Health Information System (PHIS) database from 2011 to 2017. PHIS is an administrative database containing clinical and resource utilization data for inpatient encounters from 51 children's hospitals in the United States, all of which had an ECMO program. Detailed information regarding therapies are contained within the database via Clinical Transaction Classification (CTC) codes. We included children 0 to 18 years of age, with an ECMO flag (which correlated with either an ICD9/10 code of 5A15223 or a charge mapped to CTC codes 521180, 521181 or 521182). The transfusion of all blood products and medications were identified via CTC codes (see Table 1). All data were analyzed on the encounter level, meaning that a patient who had two separate hospitalizations each involving ECMO support may be included twice. However, for patients who had more than one course of ECMO during a hospitalization, we only included the first ECMO run. We analyzed data from the first 28 days of the course of ECMO.

**Table 1:** Coding for transfusions and medications.

**Product/Medication**	**CTC Codes Queried**
Aminocaproic Acid	161,441
Cryoprecipitate	345,020
Factor VIIa	161,415
Plasma	354,041, 354,042, 354,043
Platelet	354,060, 354,061, 354,062, 354,063, 354,082, 354,083
Tranexamic Acid	161,445

### Statistical Analysis

Blood components and derivatives, and hemostatic medications were summarized by patients on ECMO for up to 28 consecutive days. Categorical variables, counts, and percentages of patients were analyzed by chi-square testing. Linear regression analysis was performed for detecting associations among transfusion/medications and time interval. Alpha was set at 0.05 for all the analyses. De-identified study data were stored in Microsoft Excel® (Microsoft Corp., Redmond, WA). Statistical analyses were performed using Stata® version 16 (StataCorp LLC, College Station, TX).

## Results

### Patient Characteristics

There were initially 9,192 children supported by ECMO identified within the database. Of these, 7,910 (86%) of the patients had complete data and were therefore included in the study cohort. Fifty-five percent of the cohort was male and the median (IQR) age was 0 (0–2) years. The patients were supported by ECMO for a total of 56,079 days. The median course of ECMO was 5 (2–9) days and the overall in-hospital mortality rate was 34%.

### Hemostatic Transfusions

**Figure 1:**
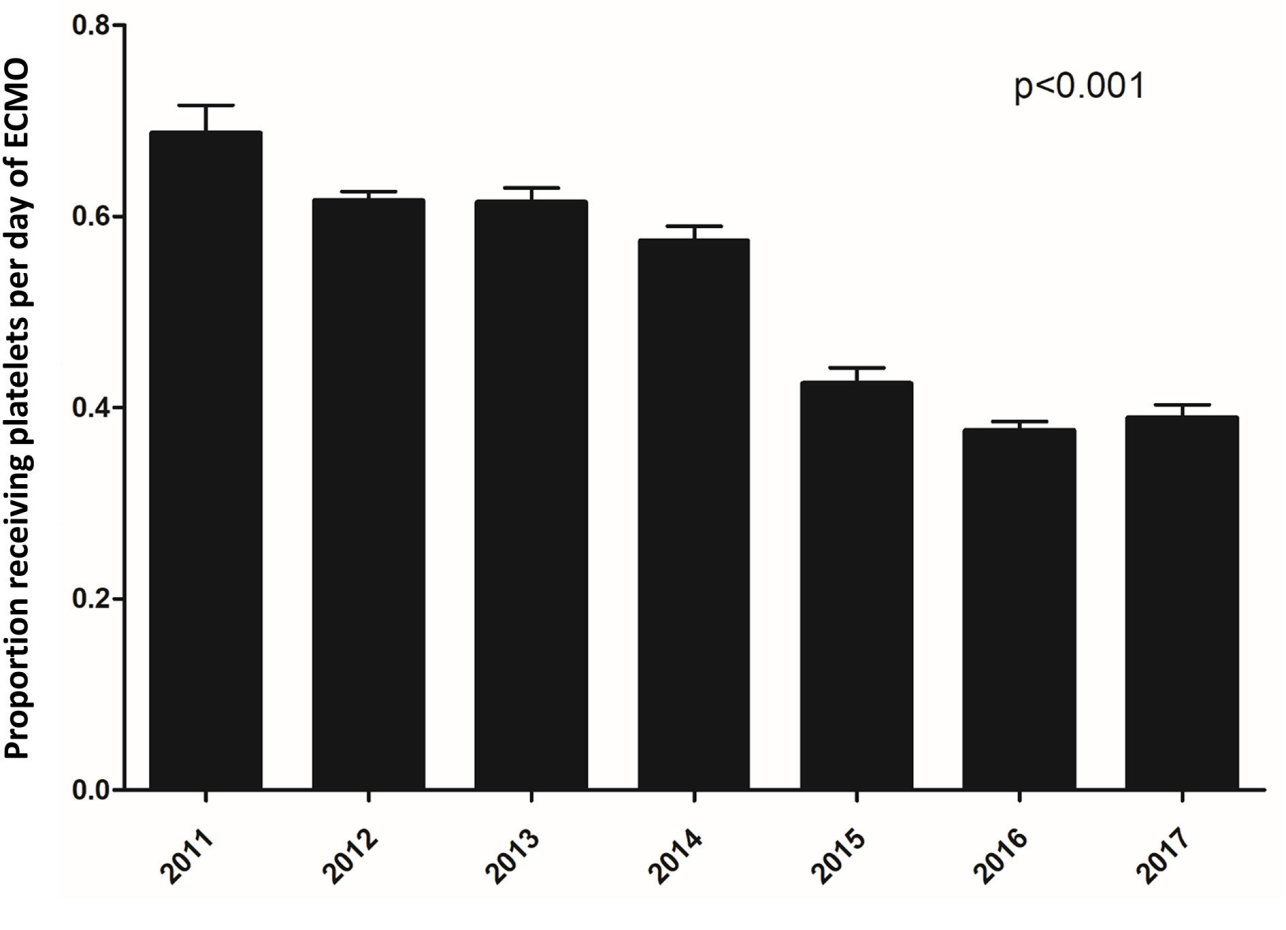
Proportion of ECMO days involving platelet transfusion from 2011–2017.

Platelet transfusions were prescribed on 49% (27,447/56,079) of ECMO days. The receipt of platelet transfusions decreased steadily over the course of the ECMO run from 57% of patients receiving a platelet transfusion on day 1 to 39% on day 28 (*p* < 0.0001). The overall use of platelet transfusions over the 6-year period of the study is depicted in Figure 1. The percentage of ECMO days with platelet transfusions decreased significantly over the study period with 69% of ECMO days involving a platelet transfusion in 2011 to 39% of ECMO days in 2017 (*p* < 0.001).

**Figure 2:**
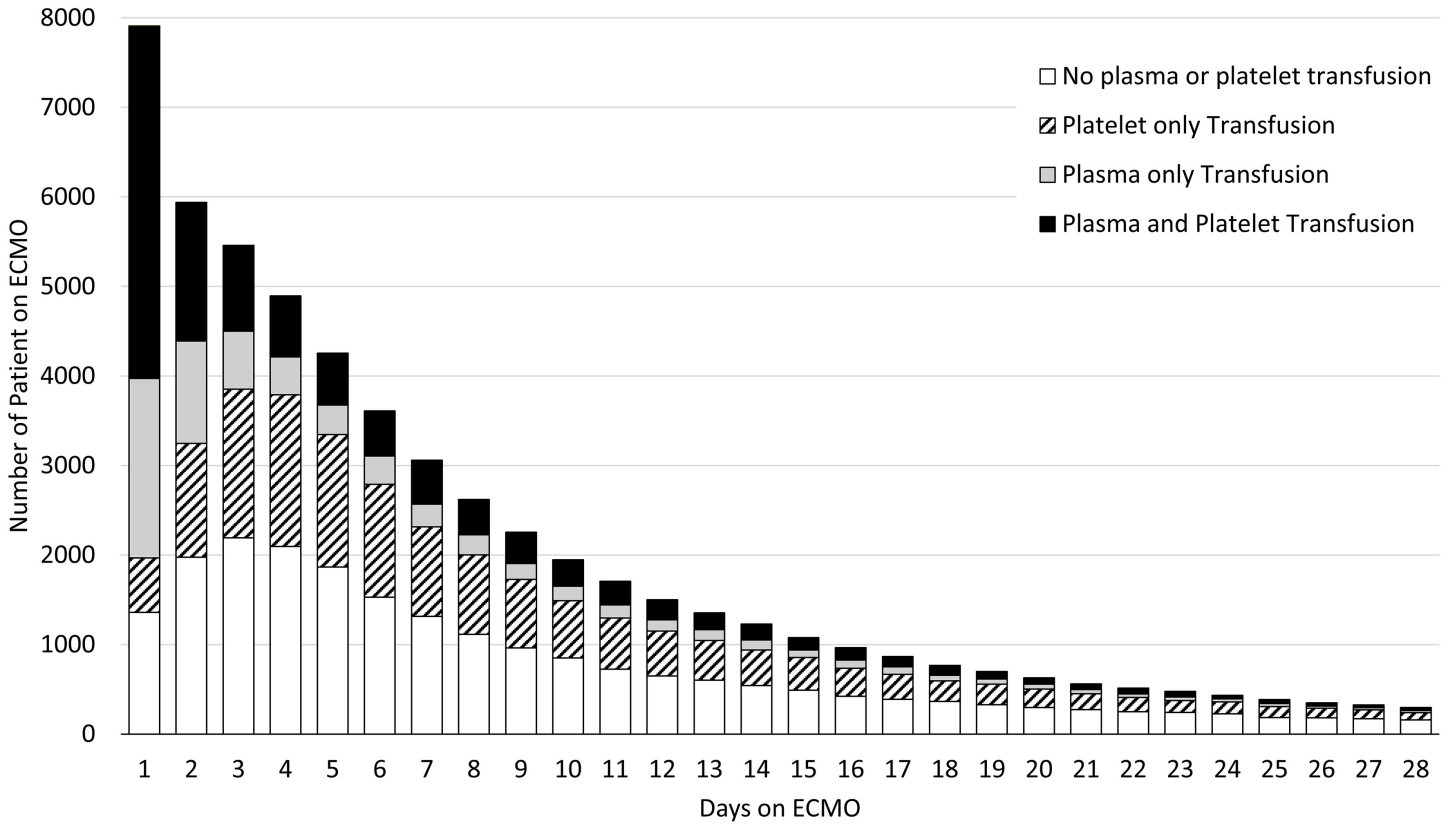
Utilization of plasma and platelet transfusions over the First 28 days of ECMO.

**Figure 3:**
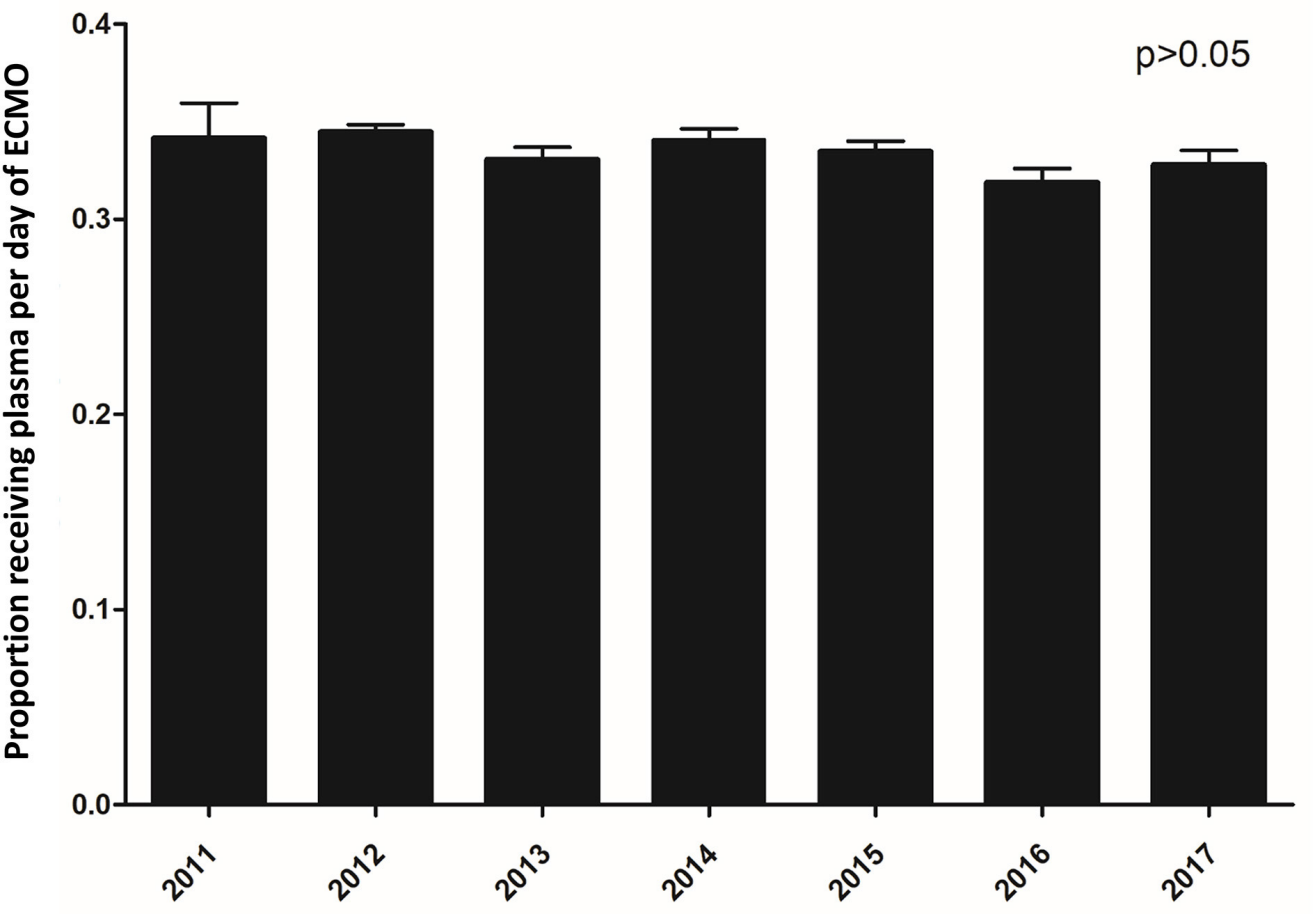
Proportion of ECMO days involving plasma transfusion from 2011–2017.

Plasma transfusions were prescribed on 33% (18,508/56,079) of the ECMO days. The receipt of plasma transfusions decreased significantly over the course of the ECMO run from 75% of patients receiving a plasma transfusion on day 1 to 20% on day 28 (*p* < 0.0001). Of note, the largest decrease in utilization of plasma occurred in the first three days. The utilization of plasma and platelet transfusions over the first 28 days of ECMO is shown in Figure 2. The overall use of plasma transfusions over the 6-year period of the study is depicted in Figure 3. The percentage of ECMO days with plasma transfusions did not vary significantly over the 6-year study period with 33% of ECMO days involving a plasma transfusion in 2011 as compared to 32% in 2017 (*p* = 0.07).

Cryoprecipitate was transfused on 17% (9350/56,079) of the ECMO days. The majority was given on day 1, with 43% of all patients receiving cryoprecipitate. The number decreased substantially (*p* < 0.001) with 20% of children receiving cryoprecipitate on day 2 and 8–14% everyday thereafter.

### Hemostatic Medications

**Figure 4:**
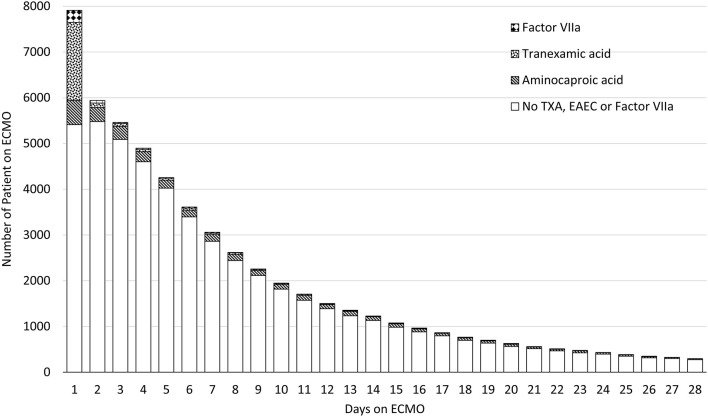
Utilization of hemostatic medications over the First 28 days of ECMO.

**Figure 5:**
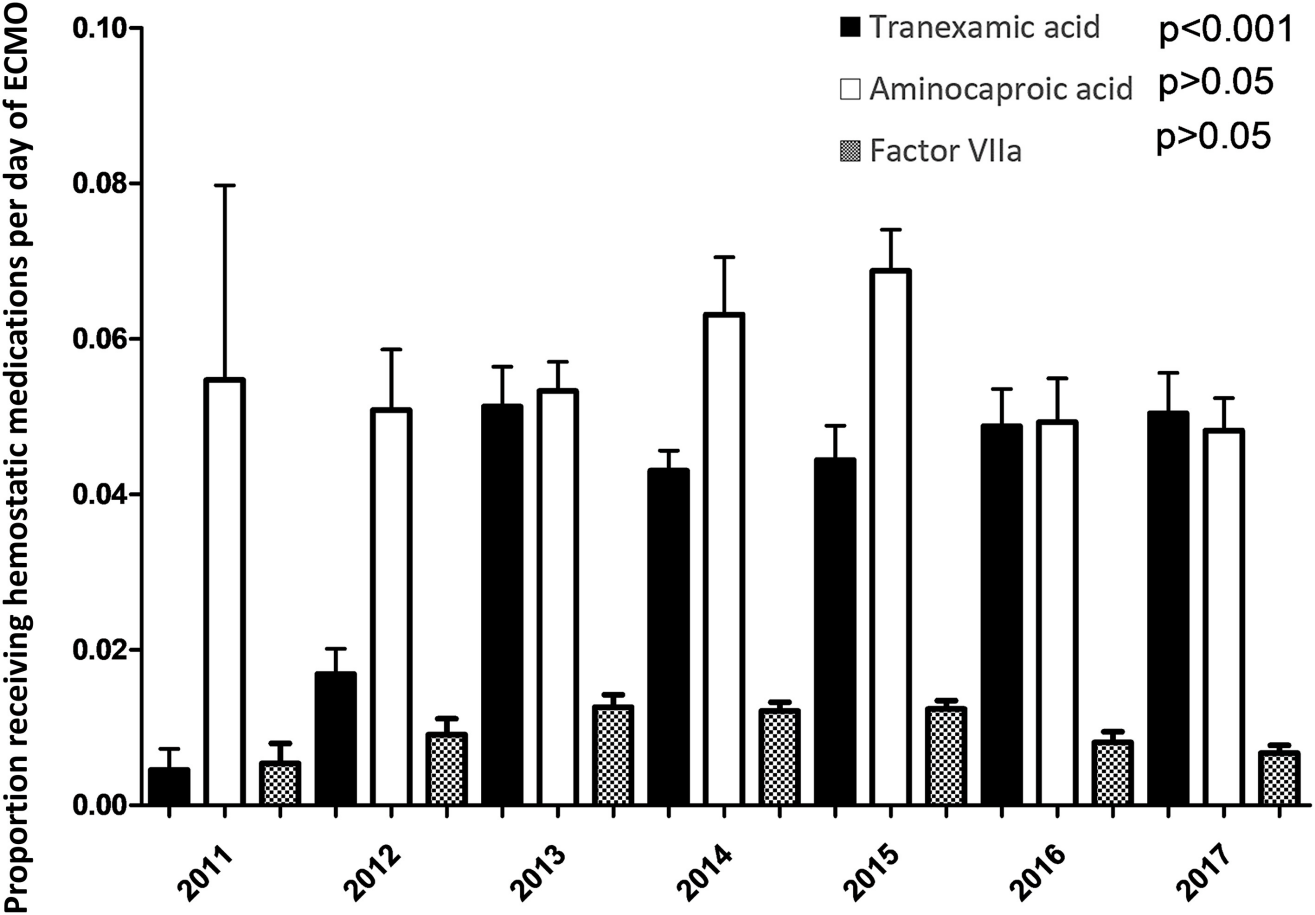
Proportion of ECMO days involving hemostatic medications from 2011–2017.

TXA was most frequently prescribed on day 1 of ECMO with 22% of all patients receiving at least one dose. Its use sharply declined after day 1 (*p* < 0.001). TXA was utilized in 4% (2294/56,079) of total ECMO days. In contrast, aminocaproic acid was prescribed evenly throughout the course of the ECMO run and used in 6% (3096/56,079) of all ECMO days. Factor VIIa was used in 1% (533/56,079) of ECMO days. The usage of hemostatic medications over the course of the ECMO run is depicted in Figure 4. The use of these hemostatic medications over the study period is depicted in Figure 5. Notably, the use of TXA increased significantly (*p* < 0.001) while there was no significant trend in the use of aminocaproic acid or Factor VIIa.

## Discussion

This PHIS cohort represents the largest group of children supported by ECMO in which hemostatic measures are described. In nearly 8,000 children, platelet transfusions were the most common and prescribed on nearly half of all ECMO days, followed by plasma on one-third of days and cryoprecipitate on one-sixth. Hemostatic medications were less common with the use of anti-fibrinolytics occurring most at the initiation of ECMO. During the 6-year study period, the prescription of platelet transfusions significantly decreased while that of TXA substantially increased.

The epidemiology of the use of hemostatic transfusions reported in this cohort is similar to findings in smaller, more detailed pediatric cohorts. In a cohort of 514 children supported by ECMO in 2012–2014, fifty-six percent received a platelet transfusion within the first 12 h of ECMO initiation (10). In a separate study of this same population, the incidence of platelet transfusion per day of ECMO (over the first 28 days) was 68% (7), slightly higher than our PHIS cohort.

Platelet transfusions are often given to children supported by ECMO due to both qualitative and quantitative platelet defects. At the initiation of ECMO, due to hemodilution, the platelet count has been estimated to drop by 25–60% in pediatric patients (11). In addition, due to shear stress, platelets are prematurely activated which leads to platelet consumption (12) and the expression of the von Willebrand receptor on the surface of platelets is reduced (13). Both of these changes can theoretically increase the patient's risk of bleeding. However, this risk must be balanced with the risk of platelet transfusions themselves. In the cohort of children supported by ECMO described above (10), each mL/kg platelet transfusions were independently associated with bleeding [OR 1.01, CI (1.00–1.01), *p* < 0.001], thrombosis [OR 1.01, CI (1.00–1.02), *p* = 0.010], and mortality [OR 1.05, CI (1.03–1.08), *p* < 0.001]. This may due to the inflammatory nature of platelet transfusions. During storage, platelets release large amounts of bioactive and pro-inflammatory substances into the storage medium, including platelet microvesicles (14). These released molecules may contribute to the morbidity associated with the transfusion of platelets. Currently, both the Extracorporeal Life Support Organization (ELSO) (15) and the American Association of Blood Banks (AABB) (16) have recommended platelet transfusion thresholds of 100 × 10^9^/L, but both groups acknowledge the recommendations are based on expert opinion and little evidence. Given the high frequency of usage of platelet transfusions in this patient population and the many associated risks, clinical trials are warranted.

Similarly, our cohort received a relatively large proportion of plasma transfusions. This finding matched that of the smaller previously published pediatric cohort in which plasma was transfused on 34% of ECMO days (7). ELSO guidelines do not directly address the use of plasma transfusions in patients who are no bleeding but do state that plasma can be given to correct factor deficiencies or to replete fibrinogen in the bleeding patient (15). And while no studies to date have examined the association between the receipt of plasma and clinical outcomes in children supported by ECMO, deleterious effects of plasma have been observed in the general population of critically ill children. In one cohort of over 800 children, the receipt of plasma was independently associated with increased risk of multiple organ failure, nosocomial infections, and longer length of stay (17). Given the use of plasma has been relatively consistent over the 6-year period of our study and the associated morbidities, the appropriate use of plasma in this patient population should also be explored.

Far less work has examined the epidemiology of the use of cryoprecipitate in critically ill children. Our findings that cryoprecipitate is transfused on approximately one-sixth of ECMO days does match previous reports (7). Significant hypofibrinogenemia/dysfibrinogenemia often occurs in patients on ECMO due to the binding of fibrinogen to the non-endothelial surface of the ECMO circuit and this has been associated with bleeding (4). Though less commonly used, fibrinogen concentrate has been shown to be associated with less allogeneic transfusions with no increased morbidity as compared to cryoprecipitate in children on cardiopulmonary bypass (18) and may be considered in children on ECMO as well.

The interaction of the circulating blood with the ECMO circuit can lead to fibrinolytic activation (19), and therefore the use of anti-fibrinolytic medications may be warranted. We report that aminocaproic acid was prescribed on 6% of ECMO days and TXA was used on 4% of ECMO days with increasing utilization over time. However, the use of these medications has been primarily studied in children on cardiopulmonary bypass. In a systematic review and meta-analysis from 2012, when compared to placebo, TXA was associated with fewer blood component transfusions, but the data was too limited to assess its effect on mortality (20). Similar results were reported with the use of aminocaproic acid in children undergoing cardiac surgery on cardiopulmonary bypass; they received less blood component transfusions and had lower rates of surgical re-exploration (21). Aminocaproic acid has also been studied in small cohorts of neonates supported by ECMO. In a 10-year retrospective study of over 400 neonates, there was no change seen in the rates of intracranial hemorrhage, but the use of aminocaproic acid was associated with decreased surgical site bleeding (22). A small randomized controlled trial in a similar patient population suggested safety in the use of aminocaproic acid but showed no difference in hemorrhagic complications (23). Given the findings in cardiopulmonary bypass, the risks associated with hemostatic transfusions, and the increased usage of antifibrinolytics, the use of TXA and aminocaproic acid for children on ECMO also deserves attention.

The study is not without limitations. As with all large database studies, granular data is missing. Most importantly, the mode of ECMO (VA vs. VV) and indication for ECMO are not delineated. While one could try to ascertain some of these details through a review of primary and secondary diagnoses, we thought these assumptions could introduce significant error into the study. The change observed in the utilization of hemostatic blood components over time may simply be a reflection of different patient indications. For example, children placed on ECMO following cardiopulmonary bypass may require more platelet transfusions due to platelet dysfunction post-operatively. Additionally, the lack of granular data prohibits an analysis to describe the independent association of the receipt of hemostatic agents and clinical outcomes since we cannot assess confounders. The indication and dosing for the hemostatic agents were not recorded and they may have been prescribed for prophylactic or therapeutic purposes. The results of laboratory assays were not reported and therefore we were unable to describe pre-transfusion values. Lastly, though large, the cohort only represents children's hospitals within the United States and may not reflect international practices, especially as many countries use fibrinogen concentrate instead of cryoprecipitate.

## Conclusions

Pediatric patients supported by ECMO are exposed to large numbers of hemostatic transfusions. Given the risks associated with the transfusion of platelets, plasma and cryoprecipitate, and the concurrent increase in the use of hemostatic medications, such as antifibrinolytic medications, studies to examine optimal strategies to prevent and treat bleeding in this vulnerable patient population are urgently needed.

## Data Availability Statement

The data analyzed in this study is subject to the following licenses/restrictions: The datasets are available to PHIS members. Requests to access this dataset should be directed to PHIS members.

## Ethics Statement

The studies involving human participants were reviewed and approved by Weill Cornell Medicine. Written informed consent from the participants' legal guardian/next of kin was not required to participate in this study in accordance with the national legislation and the institutional requirements.

## Author Contributions

MN, LV, RG and OK conceptualized and designed the study, conducted the initial analyses, drafted the initial manuscript, and reviewed and revised the manuscript. All authors contributed to the article and approved the submitted version.

## Conflict of Interest

The authors declare that the research was conducted in the absence of any commercial or financial relationships that could be construed as a potential conflict of interest.
